# The Pressurized Skin: A Review on the Pathological Effect of Mechanical Pressure on the Skin from the Cellular Perspective

**DOI:** 10.3390/ijms242015207

**Published:** 2023-10-15

**Authors:** Wei-Chen Chien, Tsen-Fang Tsai

**Affiliations:** 1 Department of Medical Education, National Taiwan University Hospital, No. 7, Chung-Shan South Road, Taipei 100, Taiwan; 2 Department of Dermatology, National Taiwan University Hospital, College of Medicine, National Taiwan University, No. 7, Chung-Shan South Road, Taipei 100, Taiwan

**Keywords:** pressure, skin cell, fibroblast, keratinocyte, mast cell, melanocyte, adipocyte, stem cell, mechanotransduction

## Abstract

Since human skin is the primary interface responding to external mechanical stimuli, extrinsic forces can disrupt its balanced microenvironment and lead to cutaneous lesions. We performed this review to delve into the pathological effects of mechanical pressure on skin from the cellular perspective. Fibroblasts of different subsets act as heterogeneous responders to mechanical load and express diverse functionalities. Keratinocytes relay mechanical signals through mechanosensitive receptors and the ensuing neurochemical cascades to work collaboratively with other cells and molecules in response to pressure. Mast cells release cytokines and neuropeptides, promoting inflammation and facilitating interaction with sensory neurons, while melanocytes can be regulated by pressure through cellular and molecular crosstalk. Adipocytes and stem cells sense pressure to fine-tune their regulations of mechanical homeostasis and cell differentiation. Applying mechanical pressure to the skin can induce various changes in its microenvironment that potentially lead to pathological alterations, such as ischemia, chronic inflammation, proliferation, regeneration, degeneration, necrosis, and impaired differentiation. The heterogeneity of each cellular lineage and subset from different individuals with various underlying skin conditions must be taken into consideration when discussing the pathological effects of pressure on the skin. Thus, elucidating the mechanotransduction and mechanoresponsive pathways from the cellular viewpoint is crucial in diagnosing and managing relevant dermatological disorders.

## 1. Introduction

The skin, as the human body’s largest external organ, is often the primary interface and responder to external mechanical stimuli. While the intrinsic mechanical forces created by the epidermal, dermal, and subcutaneous components uphold skin tensegrity, extrinsic forces may dysregulate the balanced microenvironment, leading to cutaneous lesions [1,2]. Among the mechanical forces we encounter in our daily lives, pressure stands out as an important but often overlooked physical insult. For instance, pressure injury (PI) is often mentioned when discussing the effect of pressure on skin disorders. The high intensity and extended exposure to pressure give rise to irreversible tissue ischemia of the skin and protracted inflammation during wound healing, whereas multiple pathophysiologic determinants like direct cellular damage and promotion of cellular senescence have been hypothesized to make a concerted effort to develop PI at pressure-prone body regions [3]. Given the limited evidence regarding the detailed cellular pathophysiology of pressure-related skin disorders, comprehending the cellular mechanisms of pressure’s pathological effects on the skin is of paramount importance to refine disease management. In response to pressure, skin cells and the extracellular structures relay sensed signals and integrate them via mechanotransduction, resulting in alterations in cellular morphology, functionality, and genetic profile, which may culminate in cutaneous pathologies [4]. Cells may undergo pathological deformation and damage under pressure, followed by cytoskeletal restructuring and modifications in cellular dimensions, which in turn result in tissue breakdown [5]. The reactions of different skin cells to compressive and other mechanical stresses can be distinct and unique, leading to various benign and malignant cutaneous disorders [1]. In this review, we aim to delve into the pathological effects of mechanical pressure on skin cells to improve our understanding of pressure-related skin disorders from the cellular perspective.

## 2. Methods

We conducted a comprehensive search of the literature in the electronic databases (PubMed, Embase, Google Scholar) for relevant articles from inception to 31 August 2023 with the keywords of (pressure OR compression OR weight-bearing OR gravity OR mechanical load) AND (skin OR dermis OR cutaneous) AND (“targeted cell type”, e.g., fibroblast). The titles and abstracts of articles were examined to include those focusing on how the targeted skin cell senses or responds to external mechanical pressure that potentially leads to skin lesions for review. Articles that refer to pressure as psychological stress were excluded. Articles primarily discussing the potential effects of hydrostatic or air pressure were not included. Studies focusing on the direct therapeutic effect of pressure on skin were not taken into consideration either. Only the literature available in English was included.

## 3. Results

External mechanical forces including pressure are amenable to the alterations of skin homeostasis, together with tissue architecture, and cell fate in cutaneous structures [6]. After a prudent literature review, we herein selected several types of skin cells that were more investigated in the literature and focused on the effect of pressure on these main cell lineages of the skin: fibroblasts, keratinocytes, mast cells, melanocytes, adipocytes, and stem cells.

### 3.1. Fibroblasts: Heterogeneous Responders to Mechanical Load

Fibroblasts comprise a vast proportion of mesenchymal cells in human skin and are amenable to a myriad of physiological functions and alterations of the integumentary system, such as structural support, wound repair, and skin senescence [7,8]. The heterogeneity of fibroblasts in their histological hierarchies, anatomical sites, and molecular expressions contributes to the different functions of cutaneous components [7,8]. These cells respond characteristically to mechanical stimuli through various molecules, such as leucine-rich repeats and immunoglobulin-like domains 1 (LRIG1) expressed by the papillary fibroblasts, and delta-like non-canonical Notch ligand 1 (DLK1) by the reticular fibroblasts [7,9]. During wound healing, reticular fibroblasts respond firstly to the external stress and initiate the healing process that generates scars (“scarring fibroblast”), followed by the re-epithelialization, revascularization, and regeneration of skin appendages conducted by papillary fibroblasts (“regenerative fibroblast”) [9]. The bottom-to-top order of wound repair indicates the mechanotransduction ability of fibroblasts in the skin, and the process can be further consummated through the development of contractile myofibroblasts.

**Figure 1:**
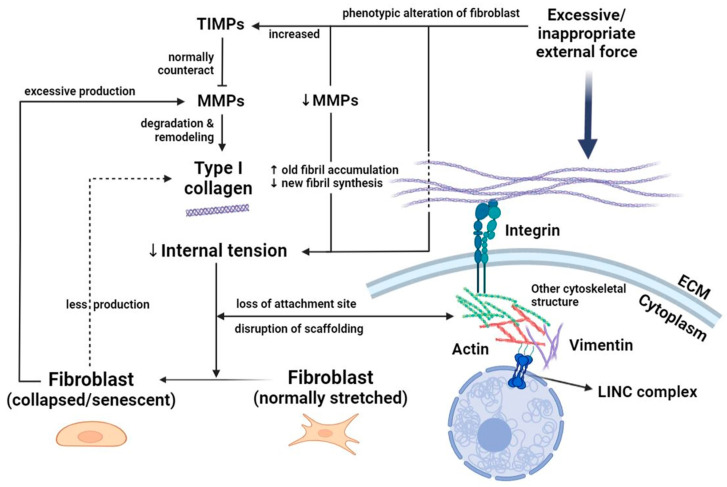
Schematic diagram of the effect of pressure on skin fibroblasts. Fibroblasts are normally stretched under the impact of intrinsic mechanical tension and provide persistent mechanical resistance of the skin through remodeling of the ECM consisting of type I collagen. External forces, including pressure, are transduced with the aid of LINC complex, integrin, and cytoskeletal structures to facilitate further responses. Once encountering excessive or inappropriate mechanical insult, fibroblasts may “collapse” due to the compromised mechanical tension followed by collagen fragmentation, detachment of integrin, and further disruption of scaffolding. Decreased production of physiologically functioning degrading enzymes (such as MMP-14) can lead to the accumulation of old collagen fibrils and the hindrance of new fibril synthesis, resulting in exacerbation of the condition. The collapse of fibroblasts stagnates new collagen production (dashed arrow) and induces excessive MMPs that dysregulate normal remodeling and impair structural integrity, thereby lapsing into a vicious cycle. ECM—extracellular matrix; LINC—linker of nucleoskeleton and cytoskeleton; MMPs—matrix metalloproteinases; TIMPs—tissue inhibitors of matrix metalloproteinases.

As a commonly used surrogate indicator of strain and stress in the dermis, myofibroblasts respond to mechanical load with the gradual incorporation of α-smooth muscle actin (α-SMA)-positive stress fibers upon signaling, while β1 integrins are of importance to bridge the interaction between the extracellular matrix (ECM) and fibroblasts, especially α11β1 [10]. Mechanical stress can be transduced through cytoskeletal structures, such as vimentin, and is relayed into the nucleus via the linker of the nucleoskeleton and cytoskeleton (LINC) complex (Figure 1) [10,11]. In addition, there is also immunologic heterogeneity among fibroblastic subpopulations of different body regions. For instance, the abundance of α-SMA detected in lesional fibroblasts of vitiligo implies their strong inclination toward myofibroblast differentiation and collagen production, which is compatible with the reduced proliferation potential and upregulated cyclic-adenosine-monophosphate (cAMP) signaling of these cells [12]. The discrepant nature of fibroblasts between diseased and non-diseased skin may potentially cause divergent effects of mechanical pressure on distinct subsets of fibroblasts in skin lesions. Fibroblasts thereby differ anatomically, immunologically, and genetically, and are influenced variously by mechanical forces and key cytokines such as transforming growth factor β (TGF-β) [8,9,10].

The renowned theory of fibroblast collapse laid the foundations for interpreting the negative effects on skin impacted by the dynamic changes in fibroblasts (Figure 1) [13]. Despite focusing on skin aging, the theory referred to the harmful result of decreased mechanical tension of the internal skin architecture. Excessive mechanical load can lead to destruction of skin tissue and reduced intrinsic mechanical tension that compromises tensional homeostasis [14]. In aging skin, fibroblasts are less contractile, proliferative, and responsive to TGF-β, and are more susceptible to oxidative stress, with matrix metalloproteinases (MMPs) being overproduced via feedback regulation that further exacerbates collagen fragmentation [15,16]. The age-related changes in the skin provide us with a pertinent viewpoint to look into the effect of mechanical pressure on fibroblasts since thinned, less resistant elderly skin can be the breeding grounds of various pressure-related skin conditions [15,17]. The dermal ECM provides mechanical strength, resiliency, and an environment that supports the functions of fibroblasts and other types of dermal cells. Thus, altered mechanical forces such as external pressure may cause ECM fragmentation and downregulate type II TGF-β receptor, which further reduces ECM production and increases ECM breakdown in a feedforward manner during aging [18] and contribute to some of the pressure-related dermatoses commonly seen in the elderly population.

Moreover, short-term mechanical stretching facilitates papillary fibroblast proliferation, ECM expansion, and subsequent skin regeneration, whereas long-term pressure results in stagnated cell growth and decreased platelet-derived growth factor receptor alpha-positive (PDGFRα+)/LRIG1+ papillary fibroblasts, causing premature senescence via cellular injury and poor replenishment of the cells [9]. This finding highlighted the duration of mechanical forces inflicted on fibroblasts being a pivotal factor that determines their character in pressure-induced dermatological disorders. Although the detailed mechanotransduction pathways are still under investigation, both the dual effects of mechanical pressure on skin fibroblasts and the heterogeneity within their population are crucial biological factors in disease development.

### 3.2. Keratinocytes: The Vanguard against Pressure

**Figure 2:**
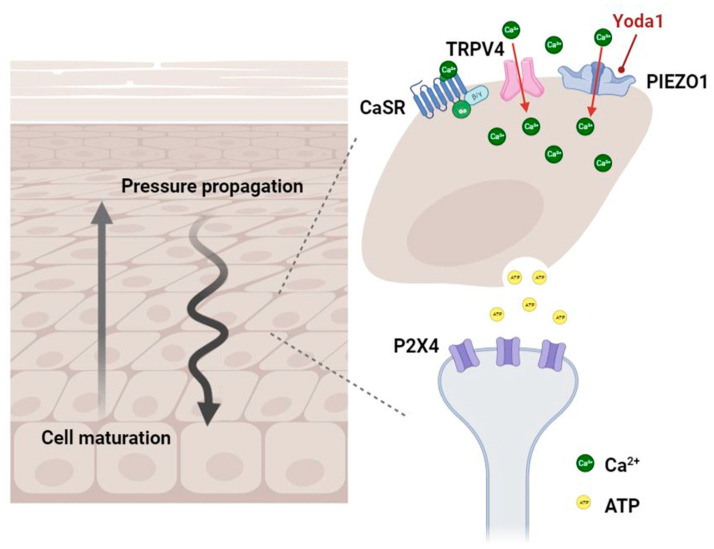
The propagation of pressure in the epidermis and certain mechanotransducers involving the modulation of mechanical stimuli in keratinocytes. The upward maturation of keratinocytes within the epidermis contributes to the mechanical resistance of skin. Among all membranous channels and receptors, CaSR, TRP (TRPV4 as representative), and PIEZO channels are major categories in charge of mechanotransduction. Yoda1 can act as an agonist of PIEZO1 channel and generates calcium influx in association with other signaling pathways, eliciting further neurochemical responses that expedite signal modulation. The release of ATP from keratinocytes also interacts with P2X4 receptors of sensory neurons to mediate cellular mechanosensitivity toward physical stimulation. ATP—adenosine triphosphate; Ca^2+^—calcium ion; CaSR—calcium-sensing receptor; TRPV4—transient receptor potential channel subfamily V member 4.

Constituting a large portion of the epidermis, keratinocytes have been elemental in the maintenance of the integrity and barrier function of human skin. In response to a variety of endogenous (genetic alterations, moisture) and exogenous (mechanical forces, skin microbiome, toxins, ultraviolet light) stimuli, these cells integrate signals and tailor their morphologies to deal with environmental stress and promote structural neogenesis [19,20]. Keratinocytes bear loads and propagate mechanical pressure through intercellular junctions to form a force-coupling network [6]. Compared to other cell types such as fibroblasts, keratinocytes boast high stiffness reflected by a 100,000-fold difference in Young’s modulus [21]. The upward fashion of cell maturation in the stratum corneum also consolidates the barrier function of the skin (Figure 2) [21].

Epidermal keratinocytes possess a diverse range of abilities related to mechanosensory perception, supported by multiple receptors and neurotransmitters. Merkel cells and Meissner’s corpuscles are major mechanoreceptors in human skin that interact with the fast Aβ fibers, while keratinocytes interconnect relatively more with the C fibers [22]. These structures mutually regulate tactile sensation, with Merkel cells and keratinocytes being in charge of rapid mechanical sensing and subtle single-celled perception, respectively [23].

Calcium-sensing receptors and transient receptor potential (TRP) channels are major categories of mechanosensitive entities that modulate pressure and other physical forces (Figure 2) [1]. The degree of calcium flux is determined by the differentiation of keratinocytes. Flawed processes in differentiation, stemming from acquired or hereditary factors, may jeopardize the homeostasis maintained by keratinocytes and epidermal integrity that contributes to hyperproliferative skin disorders. For instance, in research on pressure-related palmoplantar keratodermas (PPKs), keratin 9 (Krt9) and keratin 16 (Krt16) are two correlative subtypes of type I intermediate filament in palmoplantar epidermis regulated by inactive rhomboid protease (iRhom2). While Krt9 expressed in volar keratinocytes is crucial for terminal differentiation and physical integrity of the epidermis [24], the stress-activated Krt16 is accountable for sensing and reacting to mechanical stress and hyperproliferative conditions at pressure-bearing regions [25,26]. In rodent models, Krt9 level is drastically lowered in Krt16-null skin [26], whereas Krt16 is induced under the biallelic void of Krt9 expression [24]. The researchers extrapolated that Krt16 is a positive regulator of Krt9, and once Krt16 expression is fully defective, impairment in differentiation and functionality of palmoplantar keratinocytes might ensue, leading to the development of pressure-induced keratoderma [26].

Denda and colleagues evaluated the responses of various cell types, including differentiated and undifferentiated keratinocytes, fibroblasts, and endothelial cells, to pressure. Among them all, differentiated keratinocytes showed significant intracellular calcium influx even under low pressure and were regarded as the most sensitive lineage to mechanical pressure [23]. Hence, calcium flux and mediators such as adenosine triphosphate (ATP) and prostaglandins (PGs) may be of importance in the pathophysiology of some pressure-related skin diseases. Furthermore, P2X4 receptors, which are abundant in A- and C-fiber neurons, interact with ATP released by keratinocytes. This ATP-P2X4 signaling pathway is crucial in influencing the mechanosensitivity of keratinocytes [27]. PIEZO1, a mechanically gated non-selective cation channel, is a key mechanotransducer in the epidermis. Rodent and human keratinocytes respond to the PIEZO1 agonist of Yoda1 and generate concentration-dependent calcium influx (Figure 2) [28]. Other biomolecules like E-cadherin [29], serum response factor (SRF), and Yes-associated protein (YAP) [6,30] also possess capabilities that impact keratinocytes in terms of cell differentiation, proliferation, homeostasis, and skin inflammation upon mechanical stretching or damage. For instance, when activated, YAP may collaborate with the TEA domain (TEAD) to induce epidermal proliferation and hinder the terminal differentiation of keratinocytes [6]. On the other hand, mechanical forces that result in a confined cell–ECM area can enhance SRF activity and its actin-regulated coactivator, megakaryoblastic leukemia 1 (MAL), which modulate the expression of genes involved in cytoskeleton production and contractility, guiding the cells toward differentiation and proper division [6].

Taken together, while keratinocytes bear the brunt of external mechanical forces, including pressure, there are many more cellular apparatuses and molecular entities that aid mechanotransduction or elicit skin pathologies once the pathways are dysregulated.

### 3.3. Mast Cells: Degranulation Facilitates Neuronal Mechanotransduction

**Figure 3:**
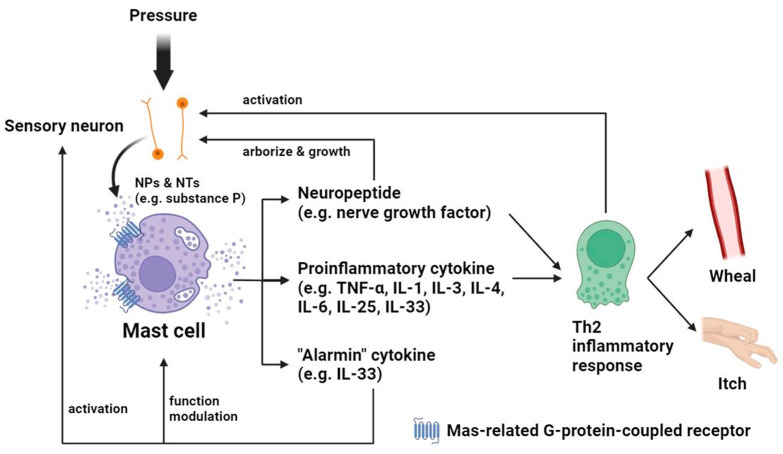
Schematic diagram of the autocrine loop of upregulated mast cell activity in urticaria in response to pressure. After the transmission of mechanical signals via neuropeptides and neurotransmitters released by sensory neurons, mast cell degranulation releases proinflammatory substances including cytokines acting as alarmin that sense tissue damage, which not only provoke Th2 inflammation but in turn modulate mast cells and activate sensory neurons. Local inflammation results in wheals and itch in urticaria patients and can activate neurons as well. Nerve growth factor, generated from mast cells during active inflammation, participates in Th2 inflammatory response simultaneously and serves as a key element in the development of local sensory nerves that affect mechanosensitivity of the skin. IL—interleukin; NP—neuropeptide; NT—neurotransmitter; Th2—T-helper 2; TNF-α—tumor necrosis factor-α.

As an effector of the T-helper 2 (Th2) immune response, skin mast cells can mediate allergic inflammatory diseases [31]. Mast-cell degranulation and inflammatory-substance release trigger sensory neuron activation and local vasodilation that drives intrinsic inflammation when encountering external pathogenic stimuli in a positive feedback manner, and these responses are particularly indispensable in the manifestations of wheals and angioedema (Figure 3) [32,33]. Mas-related G-protein-coupled receptor B2 (Mrgprb2), and its human homolog Mrgprx2 located on perineural mast cells, modulate mechanical signals through interacting with the neuropeptide substance P, affecting neurogenic inflammation and featuring a unique pathway in mast cell mechanosensitivity [32,34]. The study of acupuncture exemplifies the effect of pinpoint pressure on mast cell–neuron crosstalk, demonstrating intracellular calcium influx in mast cells upon stimulation. Neurons of perivascular distribution undergo activation via the ensuing ATP release from mast cells in the vicinity to transduce mechanical signals [35]. Moreover, an animal study suggested the compliance of mast cell distribution with the stiffness gradient of microenvironments, which could be modified by pressure. Once stiffness variations are detected, degranulation of the mast cell may be activated [36].

Local mechanical stimuli like pressure may also trigger the release of neuropeptides, such as nerve growth factor (NGF), and alarmin cytokines like interleukin-33 (IL-33) by mast cells in inflamed skin, mediating immune reactions and sensory neuron activation that further augments Th2-inflammation through the mechanical stress-induced exacerbation, leading to the presence of wheals and itch of pressure-related urticaria (Figure 3) [37].

### 3.4. Melanocytes: Cellular and Molecular Crosstalk Expedites Mechanosignaling

**Figure 4:**
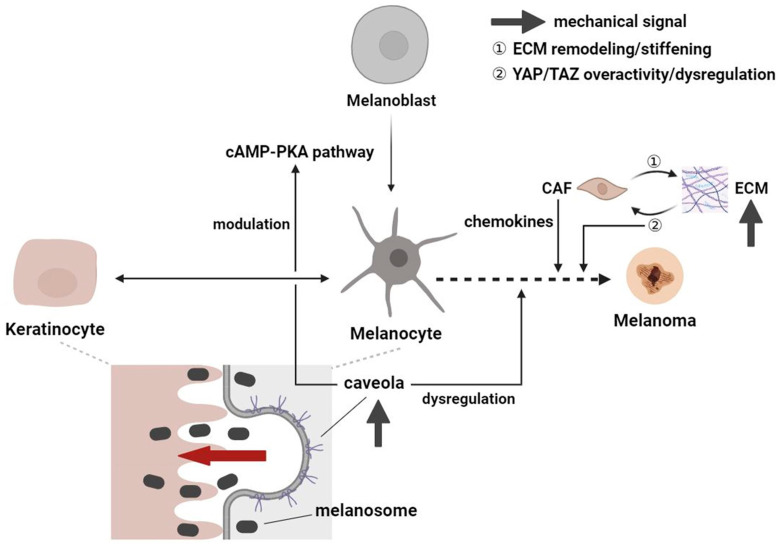
Schematic diagram of the crosstalk between melanocytes and keratinocytes and the effect of mechanical pressure on melanoma development. The differentiation of melanoblasts into melanocytes is mediated by the cAMP-PKA signaling pathway. Mechanical pressure as well as ECM stiffness may be sensed by the YAP/TAZ transcriptional cofactors and lead to the activation of cancer-associated fibroblasts, which secrete chemokines and further stiffen ECM to promote tumor angiogenesis and invasion. The transferal of melanosomes can be guided by mechanical pressure and tension, while caveolae on the melanocyte–keratinocyte interface detect and translate mechanical signals into responses, modulating the cAMP-PKA pathway to further influence melanin transfer. Dysregulation of caveolae expression can contribute to melanoma development. CAF—cancer-associated fibroblast; cAMP—cyclic adenosine monophosphate; ECM—extracellular matrix; PKA—protein kinase A; TAZ—transcriptional coactivator with PDZ-binding motif; YAP—Yes-associated protein.

Melanin production from melanocytes can be regulated by the physical and paracrine crosstalk between keratinocytes, fibroblasts, and melanocytes [12]. Factors derived from keratinocytes, including α-melanocyte-stimulating hormone (α-MSH), stem cell factor (SCF), hepatocyte growth factor (HGF), endothelin-1 (ET-1), PGE2, and PGF2α activate the cAMP-protein kinase A (PKA) pathway, through which melanoblasts differentiate into melanocytes [38]. The activation of melanocytes and mechanotransduction in response to mechanical pressure and tension may involve the transferal of melanosomes from melanocytes to keratinocytes (Figure 4) [2]. Caveolae, plasma membrane invaginations primarily situated on the keratinocyte–melanocyte interface, provide melanocytes with mechanoprotection and the translation of mechanical stimuli and secreted factors from keratinocytes into responses that affect the cAMP-PKA pathway along with the resultant melanin transfer [39]. Caveolin-1 (Cav1) constitutes caveolae majorly in terms of structure and function; once its expression is dysregulated, patients may be prone to not only melanoma but also non-melanoma skin cancer (NMSC) [40] and other hyperproliferative disorders, such as psoriasis (Figure 4) [41]. The contentious role of Cav1 as a product of oncogenes or tumor suppressor genes, as well as its dynamic change in expression level in response to mechanical signaling throughout diseases’ natural course, makes it a potential therapeutic target worthwhile to delve into [39,40].

Additionally, the YAP and transcriptional coactivator with PDZ-binding motif (TAZ) are transcriptional cofactors that play a role in the development of melanoma at weight-bearing regions [42,43]. Inputs include increased mechanical compression, interstitial pressure, inflammation, and MMP-induced ECM remodeling and stiffening from the aberrant microenvironment, giving rise to YAP/TAZ overactivity in cancerous cells, which further stiffens ECM and promotes tumor angiogenesis and invasion by cancer-associated fibroblasts (CAFs), thereby forming a positive-feedback loop that eventuates in melanoma (Figure 4) [43]. Ultrastructurally, YAP responds to the increased mechanical load of weight-bearing at the tumor margin of plantar melanoma, which leads to nuclear membrane rupture and instability as well as DNA damage in the YAP-activated cells [42]. As a mechanosensitive molecule, YAP also promotes negative durotaxis of melanoma cells following stiffness gradient changes within acral melanoma [44]. All these alterations result in tumor spread and aggression. Some investigators have examined the effects of differential gravity on the guanylyl cyclase (GC)-cyclic guanosine monophosphate (cGMP) pathway and the associated metastatic patterns in melanoma cells and melanocytes. It is proposed that under simulated microgravity, downregulated soluble GCs combined with increased expressions of natriuretic peptide-activated particulate GCs and inducible NO synthase (iNOS) may enhance tumor aggression and metastasis [45].

### 3.5. Adipocytes and Stem Cells: Versatile Navigators of Cell Fate upon Pressure

**Figure 5:**
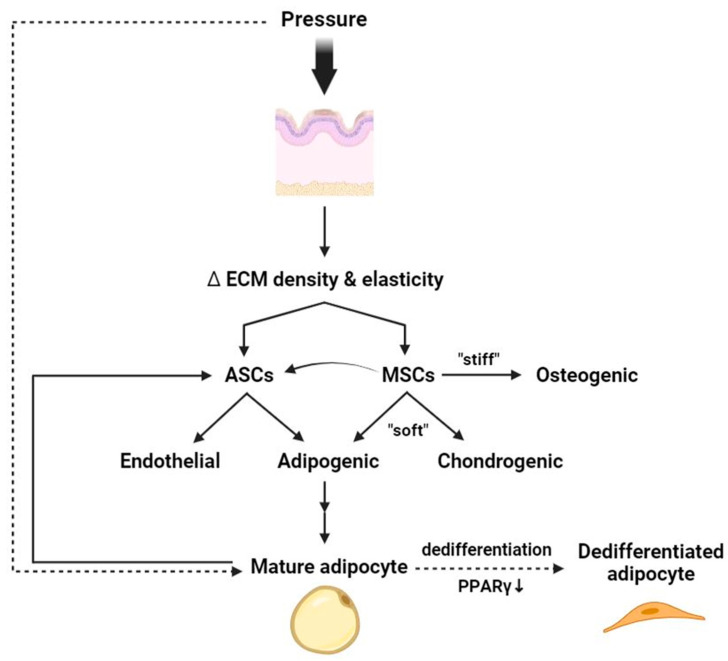
Schematic diagram of the effect of pressure on mesenchymal stem cell differentiation and adipocytes. External mechanical pressure alters ECM density, which plays a role in guiding the fate of mesenchymal stem cells (MSCs). In softer matrices, MSCs tend to differentiate into cells of adipogenic or chondrogenic lineages, while those surrounded by stiff matrix favor osteogenic differentiation. Adipocyte-derived stem cells (ASCs) are a subset of MSCs that potentially differentiate into cells of endothelial and adipogenic origins under different mechanical loading. Mature adipocytes can in turn provide mechanical cues to MSCs including ASCs that contribute to stem cell differentiation and development, while mechanical pressure may also directly provoke dedifferentiation of mature adipocytes (dashed arrows). ASC—adipocyte-derived stem cell; ECM—extracellular matrix; MSC—mesenchymal stem cell; PPARγ—peroxisome proliferator-activated receptor γ.

Aside from absorbing pressure, skin adipocytes and their precursors respond to mechanical forces through the modulation of mechanical homeostasis and cell differentiation [46]. The mechanical properties of the skin can be modulated by the subcutaneous white adipose tissue (sWAT). Perpendicular mechanical loading has the greatest impact on enhancing the stiffness of the skin/sWAT composite [47]. Since intrinsic and extrinsic mechanical signals are closely related to each other [48], compressive force creates dense ECM microenvironments, whereby YAP/TAZ activation follows focal adhesion kinase (FAK)/steroid receptor coactivator (Src) signaling, with ensuing orchestration of stem cells that determine their own hierarchies and functionalities in terms of skin homeostasis and disease development [49]. The density of the ECM, along with adipocytes and fibroblasts, provide mechanical cues to influence stem cell behaviors, ranging from proliferation and vascularization to differentiation and transdifferentiation [50,51,52]. Piroli and colleagues tested the interplay between mesenchymal stem cell (MSC) polarity and ECM stiffness, concluding that lineage specification occurred exclusively in less-stiff environments [53]. The elasticity of the ECM can steer the directionality of MSC differentiation, in which a softer matrix demonstrates an inclination toward adipogenic and chondrogenic lineages (Figure 5) [48]. Less cell spreading on the matrix and negative RhoA expression, compromising cytoskeletal tension, accelerate adipogenic differentiation, whereas, at the opposite end, osteogenesis predominates [48,54].

For adipose-derived stem cells (ASCs), different modalities of mechanical loading control the differentiation of not only the adipogenic populations but also the endothelial cells of blood vessels with regard to both morphological and functional appearances [55,56]. The effect of mechanical forces on ASCs can be modulated by oxidative stress and inflammation that leads to disparate conclusions in proangiogenic factor secretion, affecting endothelial differentiation [57]. On the other, different adipogenic cell types react to mechanical signals variously in essence, such as the contrary results in cell hypertrophy between adipocytes and preadipocytes in response to stretch [58,59]. Notably, mature adipocytes may dedifferentiate both in vivo and in vitro under mechanical pressure, implicating the self-reparation ability of the cell lineage (Figure 5) [60,61]. β1 integrin senses and transduces signals to adipocytes, in which adipogenic gene expressions involving peroxisome proliferator-activated receptor γ (PPARγ) downregulate remarkably, bringing about the smaller, multilocular, stem-cell-like morphology and regenerative potentiality of dedifferentiated adipocytes [60,61,62]. While much remains to be elucidated, the mechanics of the differentiation–dedifferentiation as well as the homeostatic regulations in adipocytes and stem cells may play a crucial role in the development of pressure-related skin disorders.

## 4. Discussion

The application of mechanical pressure can induce various changes in the skin’s microenvironment that potentially lead to pathological alterations. For instance, when the skin bears weight in a recumbent or sitting position, it may lead to an increase in local skin temperature. This can result in enhanced skin stiffness and heightened metabolic demands. Consequently, the localized skin may have reduced capability for mechanical load redistribution and may also be at risk for ischemia due to the associated increase in blood perfusion demands [63]. The development of pressure-related dermatosis is dependent on the interplay between skin cells and the mediation of molecular entities. Ischemia, chronic inflammation, proliferation, regeneration, degeneration, necrosis, and impaired differentiation are common pathological processes that can affect these cells and tissues when subjected to prolonged, excessive mechanical pressure.

In a rodent study, when the skin was exposed to compression, there was an increase in hypoxia-inducible factor-1 (HIF-1)-positive cells and thrombi formation. This was followed by the upregulation of plasminogen activator inhibitor-1 (PAI-1) due to activated HIF-1, leading to thrombosis and the subsequent development of pressure ulcers [64]. Ischemia thereby plays an important role in skin pathologies by means of direct vascular damage and HIF-1 activation following vascular occlusion in skin enduring persistent pressure. Prolonged pressure usually induces a sustained inflammatory microenvironment during the process of lesion healing [3]. While inflammation is pivotal in the mechanisms of many dermatological disorders, pressure may exacerbate the condition and ignite the development of lesions. Epidermal cells are susceptible to compressive forces and mechanical signals from the surrounding ECM, proliferating stem cells and their niches. These forces and signals promote epidermal stratification, which may contribute to the onset of hyperproliferative inflammatory skin diseases [49]. The heterogeneity of skin cells, especially fibroblasts, plays a significant role in the array of chronic inflammatory skin disorders. These differences are discernible not just in the mechanotransduction processes but also in the downstream pathomechanisms of diseases. For example, a proinflammatory effect is observed in fibroblasts expressing chemokine (C–C motif) ligand 19 (CCL19) and chemokine (C-X-C motif) ligand 1 (CXCL1) [65]. It is possible that mechanical factors may guide or influence the subset-specific pathogenic roles of skin cells to a certain degree. As our understanding of human skin at the single-cell dimension grows, functional markers and pathways like TGF-β and Notch signaling emerge as crucial regulators that promote cellular differentiation and further inflammation, presenting them as promising therapeutic targets for relevant dermatoses [65]. Another example illustrating the relationship between pressure and inflammation is seen in pressure-related urticaria, like delayed pressure urticaria (DPU). The pathogenesis usually implicates mast cells through a non-immunologic mechanism, with Mrgprb2/x2 being crucial in neuroimmunomodulation and responding to the release of proinflammatory mediators and neuropeptides by these cells [32,34,66]. These findings highlight the essential role of mechanotransduction between cellular and subcellular components in the development of skin lesions under repetitive or long-standing pressure, suggesting that oftentimes, no single cell type is involved in isolation.

The YAP/TAZ activity extensively modulates cellular proliferation, growth, differentiation, and various cell behaviors by responding to pathways such as Hippo kinase, Wnt, and G protein-coupled receptor (GPCR) signaling, and mechanical cues play a dominant role in these regulations [67]. While ECM stiffness and cell–ECM interactions guide cellular fate, YAP/TAZ plays a decisive role in regulating these processes. For instance, YAP/TAZ knockdown can promote adipogenic differentiation on stiff ECM—a process that is typically not feasible without such intervention [68]. In turn, mechanical activation of YAP/TAZ may enhance epidermal stemness by inhibiting Notch signaling, preventing epidermal stem cells from further differentiation [69]. The employment of mutant YAP2-5SA-ΔC mice also demonstrates the potential for fostering basal keratinocyte proliferation. In this context, YAP drives the Wnt/β-catenin pathway and activates the *Plau* and *Tgfbr3* genes, underscoring YAP’s significance in regenerative and neoplastic skin diseases [30,70]. Cyclic straining with mechanical pressure has been shown to modulate actin and myosin polymerization, which affects cell spreading and leads to the regulatory morphogenesis of melanoma cells. As a result, the activation of YAP in turn upregulates the expression of *NRAS* and *BRAF* genes, promoting the development of acral melanoma [71]. The increased YAP activity at the tumor margin of acral melanoma may result from the increase in ECM stiffness, which can be influenced by the cumulative production of ECM components by the infiltrated fibroblasts [42]. This finding highlights the role of fibroblasts in the mechanical process of pathological melanoma cell proliferation, beyond the known keratinocyte–melanocyte crosstalk, and suggests their contribution to cancer development. Additionally, if epidermal stem cells are deficient in the inhibitor of plexin-B, the inhibition of YAP would be compromised, resulting in the cells’ inability to sense mechanical pressure, which further disinhibits YAP [72]. This disinhibition can lead to cellular proliferation, potentially contributing to the development of basal cell carcinoma (BCC) [72]. In an in vitro study utilizing spiny mice (Acomys cahirinus) applied to human fibroblasts, treatment with verteporfin, a nuclear YAP-TEAD inhibitor, led to persistent enhancement of myofibroblast differentiation mediated by TGF-β, resulting in tissue fibrosis rather than regeneration; conversely, forcibly increasing YAP activity might prevent and rescue this process [73]. The versatility of YAP/TAZ as a mechanosensitive and mechano-modulatory molecule may, thus, be influential in a wide range of pressure-induced dermatoses.

Clinically, the fibroblastic and myofibroblastic proliferations underlie the reactive mechanisms elicited by pressure in some pressure-related skin disorders, such as the cyclist’s nodule [74]. Comparably, if local ischemia occurs under long-standing pressure, the repetitive tissue damage and regeneration can result in the proliferation of atypical fibroblasts and the subsequent development of diseases like atypical decubital fibroplasia [75]. Mechanical cues and their effects on the skin microenvironment play a crucial role in modulating fibroblast behaviors and simultaneously offer potential therapeutic targets for lesion management, such as preventing wound scarring and impacting tumorigenesis by depleting FAK and subsequently disrupting stromal forces [76]. The mechanical coupling of external forces with FAK modulates tissue-level forces and cellular migration, impacting mechanotransduction and tissue repair. Talin and vinculin aid this process, as focal adhesions interact with them to mediate the cell–ECM interplay [77]. The framework may be applied to the evolution of stem cell lineages responding to ECM stiffness and the development of biomaterials, which are prospective aspects of regenerative medicine [77]. In patients with PPKs associated with mechanical load, the upregulation of Krt9 expression in keratinocytes using agents like glycogen synthase kinase (GSK)-3β inhibitors may provide insights into potential therapeutics for this currently incurable disease entity [26,78]. Apart from being the culprit or accomplice for a multitude of skin disorders, pressure, along with other mechanical forces such as lateral compression and friction due to their frequent coexistence, may be promising targets for a therapeutic breakthrough in dermatology. Pressure offloading directly and intuitively reduces the mechanical burden on the skin, potentially sparing patients from unnecessary treatments that may not benefit them. For example, using a total contact cast (TCC) may reduce local inflammation and edema in patients with diabetic foot ulcers, a condition in which mechanical pressure can exacerbate the lesion [79]. While these designs have potential applications for a broader range of skin disorders primarily caused by pressure, the effects of offloading on skin cell changes and the skin microenvironment need further study to drive technical advancements.

Last but not least, although the pathological effect of pressure might be necessary, it may not be sufficient on its own for the development of pressure-related skin disorders. Specifically, in the context of pressure ulcers, host factors—intrinsic and unique to each individual—play a significant role in determining lesion onset [80]. Factors such as tissue tolerance to damage and varying cellular demands are crucial [80]. Given this, a deeper understanding of cellular mechanotransduction and the skin’s response to pressure from the cellular perspective may enhance clinicians’ ability to discern the effects of pressure on different patients with various relevant dermatological disorders.

## 5. Conclusions

While skin endures mechanical pressure for protection, the omnipresent extrinsic force may lead to a series of intricate alterations in the skin cells and the microenvironment. Cellular responses and interactions, particularly involving cells such as fibroblasts, keratinocytes, mast cells, melanocytes, adipocytes, and stem cells, may expedite the propagation and translation of extrinsic mechanical signals of pressure. When exposed to sustained or prolonged pressure, these interactions and responses can result in skin pathologies, and molecules like YAP are crucial in modulating and aiding these processes. Under pressure, skin cells and tissue may mechanistically undergo pathological events such as ischemia, chronic inflammation, proliferation, regeneration, degeneration, necrosis, and impaired differentiation. The heterogeneity of each cellular lineage and subset from different individuals with various underlying skin conditions must also be taken into consideration when discussing the pathological effects of pressure on the skin. Thus, further investigations of the mechanotransduction and mechanoresponsive pathways from the cellular viewpoint are of paramount importance. This will help recognize the pathophysiologies of relevant pressure-related dermatoses and tailor cellular- and molecular-oriented management.

## Data Availability

No new data were created or analyzed in this study. Data sharing is not applicable to this article.
